# Inversion Effect of Hand Postures: Effect of Visual Experience Over Long and Short Term

**DOI:** 10.1177/20416695221105911

**Published:** 2022-06-14

**Authors:** Weidong Tao, Zhen Xu, Dongchi Zhao, Chao Wang, QiangQiang Wang, Noah Britt, Xaoli Tao, Hong-jin Sun

**Affiliations:** 117774 Department of Psychology, School of Teacher Education, Huzhou University, China; The Key Laboratory of Brain Science and Children's Learning of Huzhou, Huzhou University, Huzhou, China; 117774 Department of Psychology, School of Teacher Education, Huzhou University, China; 117774 Department of Psychology, School of Teacher Education, Huzhou University, China; 117774 Department of Psychology, School of Teacher Education, Huzhou University, China; 117774 Department of Psychology, School of Teacher Education, Huzhou University, China; 3710 Department of Psychology, Neuroscience & Behaviour, McMaster University, Canada; 117774 Department of Psychology, School of Teacher Education, Huzhou University, China; 3710 Department of Psychology, Neuroscience & Behaviour, McMaster University, Canada

**Keywords:** same/different paradigm, object identification, training paradigm, expertise hypothesis

## Abstract

Some researchers argue that holistic processing is unique to face recognition supported by the face inversion effect. However, findings such as the body inversion effect challenge the face processing-specificity hypothesis, thus supporting the expertise hypothesis. Few studies have explored a possible hand inversion effect which could involve special processing similar to the face and body. We conducted four experiments to investigate the time course and flexibility of the hand posture inversion effect. We utilized a same/different discrimination task (Experiments 1 and 2), an identification task (Experiment 3), and a training paradigm involving the exposure of different hand orientations (Experiment 4). The results show the hand posture inversion effect (with fingers up as upright orientation) was not initially observed during the early phase of testing, but occurred in later phases. This suggests that both lifetime experience and recent exposure affect the hand posture inversion effect. We also found the hand posture inversion effect, once established, was stable across days and remained consistent across different tasks. In addition, the hand posture inversion effect for specific orientations could be obtained with short-term training of a given orientation, indicating the cognitive process is flexible.

## Introduction

The face inversion effect is a phenomenon whereby people demonstrate a worse performance for the identification of inverted faces compared to upright faces (Freire et al., 2000; Kilgour & Lederman, 2006; Yin, 1969). The face inversion effect is regarded as a marker for the specialized holistic processing of faces (Farah et al., 1998; Maurer et al., 2002). This phenomenon distinguished the difference between the processing mode of face recognition and that of general object recognition, supporting the face processing-specificity hypothesis (Byatt et al., 2001; Henke et al., 1998; Itier et al., 2006). In particular, upright faces are processed by holistic processing mechanisms, in which upright faces were identified via the representation of interrelations of different parts and the overall characteristics of those faces (Farah et al., 1995; Maurer et al., 2002; Tanaka & Farah, 1993), instead of individual components in the faces. However, when inverted, the configural representation of faces was impaired, making it impossible for people to use holistic processing to quickly recognize inverted faces. As for general objects, such as houses and airplanes (Yin, 1969), there is no significant inversion effect, likely due to the involvement of feature detection processing. The inversion paradigm has been widely used in the field of object recognition to compare the processing of face and nonface objects.

In addition to the face, the inversion effect has also been discovered in some nonface objects. For example, the inversion effect was shown when dog experts were asked to identify pictures of dogs (Diamond & Carey, 1986), Greeble experts who underwent specialized training were presented with Greeble (Ashworth et al., 2008; Gauthier et al., 1998; Gauthier & Tarr, 1997), and experts who were trained to distinguish houses (Husk et al., 2007) were asked to recognize various housing structures. The findings from those studies contradicted *the face processing-specificity hypothesis*, arguing that holistic processing could be used to recognize nonface objects, which was better explained by the *expertise hypothesis* (Diamond & Carey, 1986; Gauthier & Tarr, 1997; Gauthier et al., 1999, 2000). The expertise hypothesis states that even general objects can be holistically processed, with the assumption that individuals should have comprehensive experience with the objects with high within-class similarity.

Reed et al. (2003) used body postures as stimuli to investigate whether body posture recognition was similar to that of faces and found that human body posture identification was more impaired when inverted compared to that of other objects such as houses. This result suggested that participants might apply configural processing, likewise to faces, to recognize human body postures. Other studies have found similar results using different types of body stimulus materials, including gray-scale figures (Arizpe et al., 2017; Brandman & Yovel, 2010; Minnebusch et al., 2009; Yovel et al., 2010), point-light sequences (Chang & Troje, 2009; Troje & Westhoff, 2006), and 3D human body posture figures (Tao & Sun, 2013; Tao et al., 2014). Moreover, event-related potential (ERP) investigations for inverted body postures also revealed similar changes in the N170 component compared with inverted faces (Mohamed et al., 2011; Stekelenburg & de Gelder, 2004; Tao et al., 2014), which supports the idea that the processing of body postures is the same as that of faces.

Several previous studies have suggested that the head plays a special role in the body inversion effect. However, the results of studies involving head manipulations were not consistent (Mohamed et al., 2011; Tao et al., 2014; Yovel et al., 2010). According to an ERP study (Minnebusch et al., 2009), electrophysiological results showed that the inversion effect of bodies without heads was opposite to that with heads. Specifically compared to the upright bodies without heads, inverted ones revealed the improved performance and elicited a reduced N170 amplitude. Yovel et al. (2010) suggested that the head has a special status in body posture identification, and the body inversion effect is decreased for headless bodies. In contrast, Tao et al. (2014) showed that both the whole body and the piecemeal body (without head and trunk) generated a significant inversion effect. This illustrates that while the findings of previous research on the role of the head were not always consistent, the whole-body posture inversion effect was stable across different tasks, stimuli, and participants.

In the field of object recognition, one of the controversies is whether the processing modes of upright faces and inverted faces differ in quality or quantity. Some studies (Rossion, 2008) argue that the processing mode of upright faces is qualitatively different from that of inverted faces, while some others state that the processing modes are only quantitatively different. Sekuler et al. (2004) found that, after the inversion, face recognition also involves configural processing, indicating that the two processing methods are not completely opposite, but are primarily selected based on different kinds of information. Reed et al. (2006) conducted experiments to explore the type of configural processing that was adopted during body posture identification and the involvement of the configural processing continuum. They expanded the idea of the configural processing continuum to explain the body inversion effect. Reed et al. (2006) argued that on one end of the configural processing continuum, most objects (such as houses) are processed by part recognition (feature detection processing); while on the other end, other objects (such as faces) are processed via configural processing. In other words, the configural processing and feature detection processing are not diametrically opposed; they are only quantitatively different. Furthermore, the processing mode involved is determined by the type of information available. Tao and Tao (2018) also examined whether the processing mechanisms for body postures differ in quality or quantity and discovered that the body inversion effect is the result of a quantity continuation.

The human hand is a unique part of the human body. Similar to body posture, hand postures also include multiple components connected through a fixed spatial relation. Due to biological constraints, each joint allows for only a certain range of motion. In addition, humans have extensive visual exposure to both body and hand postures and possibly develop perceptual expertise in recognizing upright body postures and possibly, although to less degree, upright hand postures.

However, experience with hand posture can be different from that of body posture. For body postures, humans have far more experience with upright body postures and have little experience with inverted body postures. For hand posture, although upright hands (at least for their own hands) are exposed more often than inverted hands (thumb-down hand postures), the difference in experience for upright and inverted hand postures might not be as large. Secondly, components of body posture are more different because the head, limbs, and trunk are all very different in shape while hand posture is made up of the palm and five fingers with a similar shape. Moreover, body postures (Arizpe et al., 2017; Brandman & Yovel, 2010; Reed et al., 2003; Tao & Sun, 2013; Yovel et al., 2010) are typically hard to be named while hand postures (Gunter & Bach, 2004; Hadar & Pinchas-Zamir, 2004) are often more symbolic and convey certain meanings in human culture.

To date, while there have been isolated studies on mental rotation of hand stimuli (Tao et al., 2009a, 2009b; Thayer et al., 2001), few studies have examined whether hand posture recognition elicits the hand inversion effect. In the current study, we investigated whether there was a hand posture inversion effect. Based on the similarities between hand posture and body posture, we hypothesized that hand posture recognition, like body posture recognition, could elicit an inversion effect. Given the difference in the amount of exposure for different-hand orientations (upright versus inverted) is not as large as that of the body, we expect that any inversion effect for the hand stimuli would not be as large. In addition, given that participants might develop a strategy during the experiment, configural processing, which could be a more efficient way to process information, might be developed after some exposure to the task. Thus, we hypothesized that if in the earlier phase of the test, the hand posture processing could not elicit the inversion effect, then with an increase in exposure to the task during the experiment, participants may gradually be able to reveal an inversion effect. This would be because participants retrieve their own canonical representation of the hand postures acquired from lifetime experience. In other words, in the course of the experiment, participants might attempt to adjust the processing mode over time, leading to the hand posture inversion effect after repeated exposure. We thus used a block design to explore the time course of the hand posture inversion effect in a same/different discrimination task in Experiment 1 (5 blocks) and Experiment 2 (the same 5 blocks repeated over three days), and in an identification task in Experiment 3. During testing, we provided participants with the same degree of exposure for upright and inverted hand postures to isolate the effect of lifetime exposure to different-hand orientations. Furthermore, we trained two groups of participants to process upright or inverted hand posture to investigate whether the participants would produce an inversion effect contingent on the specific training orientation (Experiment 4).

## Experiment 1

The same/different tasks were used to investigate whether hand posture recognition would elicit an inversion effect for untrained participants. In addition, we used a block design to explore the effect of repeated stimulus presentations.

### Method

*Participants*. Sixteen undergraduate students from Southwest University in China (6 men and 10 women, average age of 20.7 years) took part in Experiment 1, and each received RMB 10 Yuan after the experiment. All participants were healthy and right-handed and had normal or corrected-to-normal vision. The study was reviewed and approved by the Institutional Review Board of the Human Research Ethics Committee of the University.

*Stimuli*. All hand postures were created using 3D Poser™ software, based on the hand model of an adult male. This software allows manipulations of different joints of the hand in the 3D model. Two types of hand postures were created. The first were biomechanically possible hand postures, limited by biomechanical constraints (Petit et al., 2003; Petit & Harris, 2005). According to the movable range of the human hand (Rothsteine et al., 2005), various hand postures were created by adjusting the joints. Specifically, we used the Use Limits function in Poser software to keep all the joints of the hand posture in the 3D model within the movable range of the normal hand. The second were biomechanically impossible hand postures that exceeded the biomechanical constraints (at least two of the joints were adjusted beyond the range). To implement this, we removed the Use Limits function in Poser software. None of the hand postures had any particular meaning.

**Figure 1.**
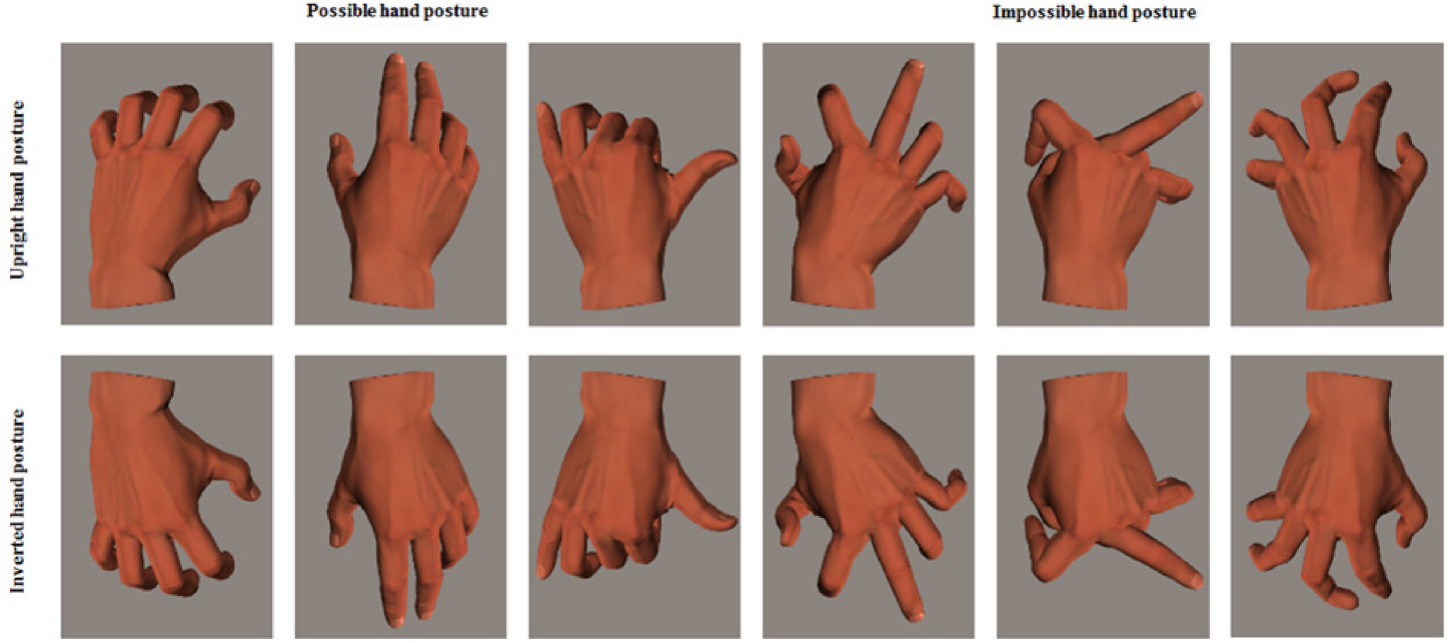
Examples of hand posture stimuli. The left six and the right six are different in biomechanical possibility; the top six and the bottom six are different in orientation.

Forty left-hand postures were created in this manner: 20 biomechanically possible and 20 biomechanically impossible hand postures. We compiled the experimental material evaluation procedure using E-Prime 1.1 software to assess the 40 left-hand postures. The stimuli were assessed by three experimenters. From the 40 left-hand postures, we selected five left-hand postures that were considered biomechanically possible and five left-hand postures that were considered biomechanically impossible. Then, we created mirror images of 10 left-hand postures using the mirror image function in Photoshop. As a result, the experimental materials were divided into 10 biomechanically possible hand postures (five left-hand and five right-hand postures, the latter being the mirror images of the former) and 10 biomechanically impossible ones (five left-hand and five right-hand postures, the latter being the mirror images of the former). Therefore, each type of hand posture (biomechanically possible and impossible) had a total of 10 pictures as formal experimental materials (see examples of three-hand stimuli in Figure 1).

Each type of hand posture was displayed in a match trial (identical-hand pairs) and a mismatched trial (different-hand pairs) from an upright or inverted orientation. Under the identical-hand condition, the second stimulus was identical to the first stimulus. Under the different-hand condition, the second stimulus was different from the first stimulus, that is, the second stimulus was created by shifting the positions of two joints of the first stimulus shown just before.

*Experiment apparatus*. The experimental materials of the hand posture evaluation program were compiled using E-Prime 1.1. The formal experimental program was compiled using Experiment-Builder l.4. The monitor was 17″ in size, with a refresh rate of 100 Hz and a resolution of 1,024 × 768. The size of the stimuli image was 400 × 300 pixels, while the height of the hand posture in the upright and inverted orientations was around 240 pixels with a visual angle <5°.

*Experiment design.* The design of the experiment was a 5 (block: 1, 2, 3, 4, 5) × 2 (match: matched and mismatched pairs) × 2 (types of hand postures: biomechanically possible and biomechanically impossible) × 2 (orientation: upright and inverted hand postures) factorial design. The experiment was composed of five blocks, with 400 trials in total. Two pictures of hand postures were presented sequentially in each trial. The order of the stimulus presentation, types of hand postures, and orientations were completely randomized in each block. In each block, half of the trials were identical-hand pairs (matched pairs), and the other half were different-hand pairs (mismatched pairs). However, only data from the same pairs (matched pairs) in the formal experiment were analyzed.

### Procedure

**Figure 2.**
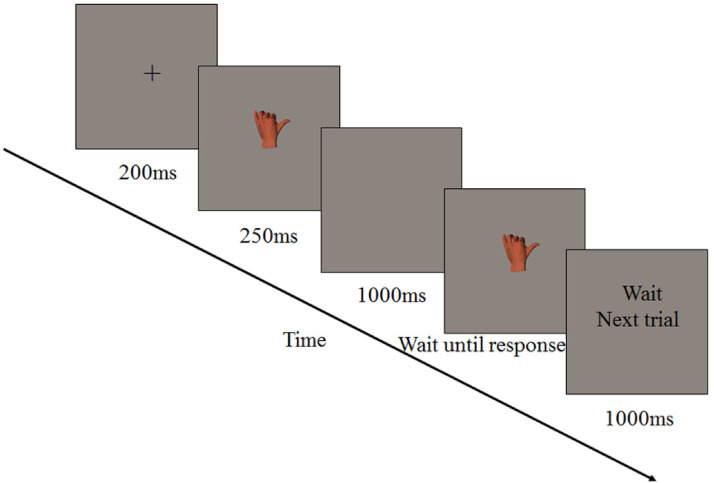
The sequence of events in a trial in Experiment 1.

Each participant was seated at a distance of 75 cm from the monitor. The center of the computer screen was placed at eye level by adjusting the height of the chair. The chin rest was used to place the jaw, the height of which was 25 cm. Each trial began with a fixation for 200 ms, followed by a stimulus lasting 250 ms. A blank screen was then presented for 1,000 ms. Next, the second stimulus was presented; it remained until the participant responded, followed by the reminder of “wait next trial” display lasting for 1,000 ms (see Figure 2). In addition, both stimuli were presented in the center of the screen and in the same orientation, either upright or inverted. Participants were instructed to press the “1” key with their right index finger when the two hand postures were identical and the “2” key with their right index finger when the two hand postures were different. Participants were required to operate per the instructions and respond as quickly as possible without compromising accuracy. Both error rates and reaction times (RTs) were recorded. Eight practice trials were conducted before the formal experiment. The entire experiment took approximately 45 min.

### Data Analysis

The error rates and RTs were analyzed. However, only the data for identical pairs (matched pairs) were analyzed, and the data for different pairs (mismatched pairs) were removed. RTs beyond three standard deviations from the mean were discarded (0.3% of trials were discarded). The results were analyzed using a 5 (block: 1, 2, 3, 4, 5) × 2 (orientation: upright and inverted hand postures) × 2 (type of hand postures: biomechanically possible and biomechanically impossible) three-way repeated-measures analysis of variance (ANOVA).

### Results

**Figure 3.**
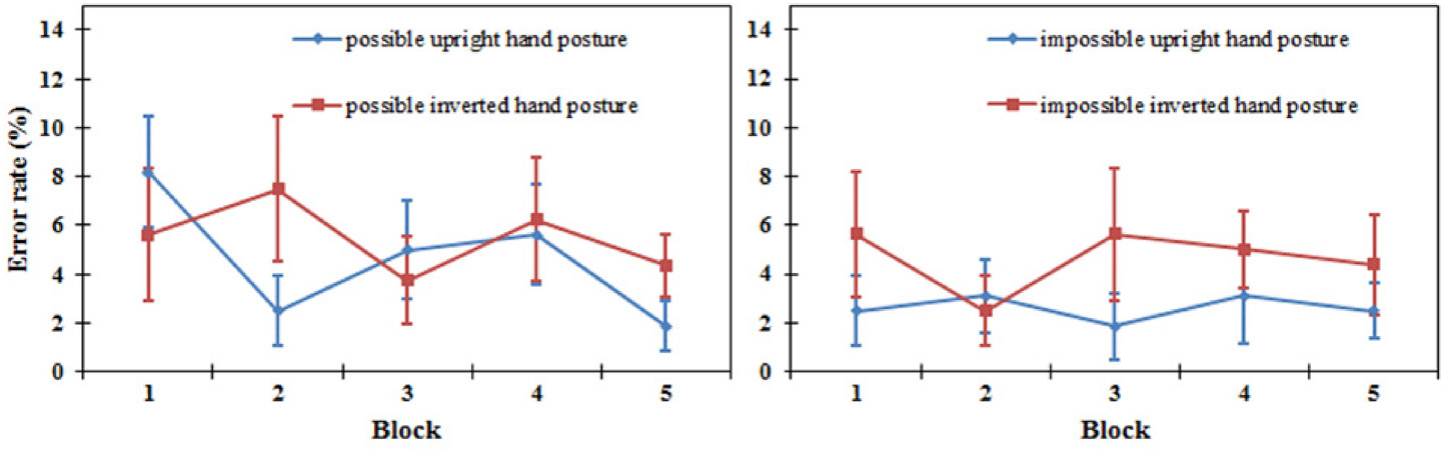
Error rates of the upright and inverted conditions in five blocks for the biomechanically possible hand postures (left) and biomechanically impossible hand postures (right) in the same/different task in Experiment 1. Error bars represent the standard errors of the means for each condition.

*Error rate*. A three-way repeated-measures ANOVA showed a significant main effect of orientation [*F*(1, 15) = 11.322, *P* *<* .01], which showed a lower error rate for upright hand postures (3.6%) compared to inverted ones (5.1%). In contrast, there was no main effect of block and type of hand posture [*F*(4, 60) = 1.003, *P* = .413; *F*(1, 15) = 2.417, *P* = .141, respectively]. No two-way interaction effects were observed. The three-way interaction effect between the blocks, types of hand postures, and orientation did not reach significance [*F*(4, 60) = 2.134, *P* = .088] (see Figure 3).

*Reaction time*. A three-way repeated-measures ANOVA revealed a significant main effect of orientation [*F*(1, 15) = 4.773, *P* < .05], which showed that upright hand postures were elicited in shorter RTs (684 ms) than inverted ones (701 ms). The main effect of blocks was significant [*F*(4, 60) = 7.033, *P* < .001], while the types of hand postures reached a marginal level of significance [*F*(1, 15) = 4.537, *P* = .050]. The two-way interaction effect between the block and orientation was significant [*F*(4, 60) = 2.715, *P* = .038], whereas the other interaction effects were not significant.

**Figure 4.**
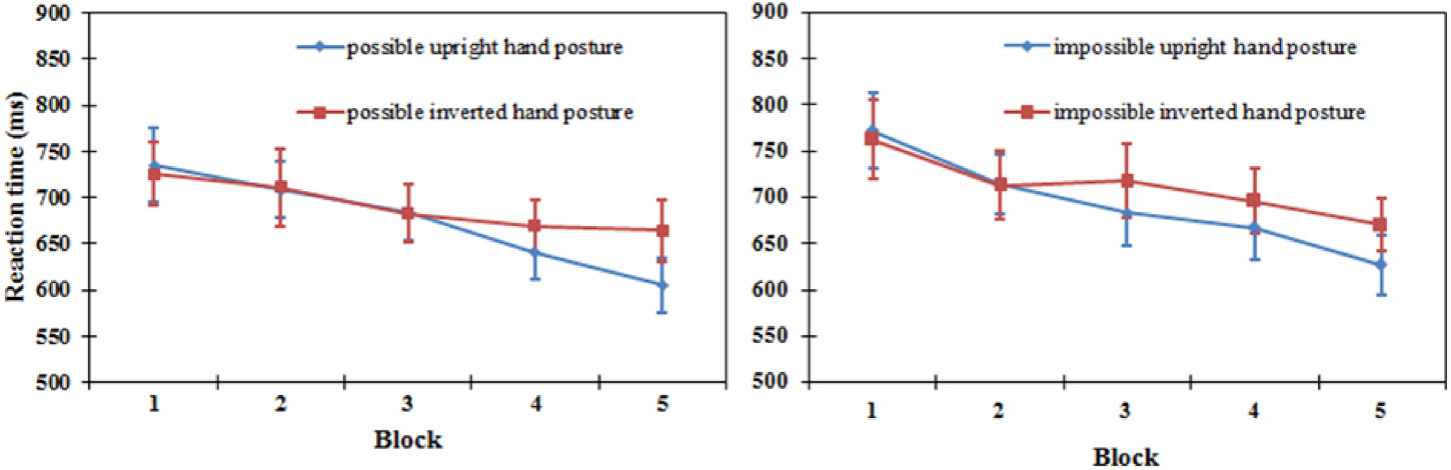
Reaction time in upright and inverted orientation in five blocks for the biomechanically possible hand postures (left) and biomechanically impossible hand postures (right) in the same/different task in Experiment 1. Error bars represent the standard errors of the means for each condition.

Further results revealed that, for biomechanically possible hand postures, the difference between upright hand postures (675 ms) and inverted hand postures (690 ms) did not reach significance [*F*(1, 15) = 3,103, *P* = .099]. For biomechanically impossible hand postures, there was no significant difference between upright hand postures (693 ms) and inverted hand postures (712 ms) [*F*(1, 15) = 1.936, *P* = .184]. Given the significant interaction between block and orientation, the test of differences was conducted with the upright and inverted orientations within each block. For biomechanically possible hand postures, only the differences between upright hand postures and inverted hand postures in blocks 4 and 5 reached significance [*F*(1, 15) = 6.02, *P* < .05; *F*(1, 15) = 9.58, *P* < .01, respectively]. For biomechanically impossible hand postures, only the difference between upright and inverted hand postures in block 5 was significant [*F*(1, 15) = 5.99, *P* < .05] (see Figure 4).

### Discussion

Adopting human hand postures as the stimuli and using a block test design, Experiment 1 was designed to investigate whether hand posture recognition was similar to body posture recognition and whether it could generate an inversion effect. The results show that for both biomechanically possible and impossible hand postures, there was no inversion effect in earlier blocks. However, as the experience accumulated, the RTs in block 5 showed that both biomechanically possible and impossible hand postures produced significant inversion effects. Overall, the results showed a progression from the lack of an inversion effect to a robust inversion effect throughout the experiment, indicating the processing mode switched from the initial prioritization of feature detection to prioritizing configural processing. Thus, the results further support the view that orientation-specific experience is a crucial element in generating object discernment inversion effects. We also found that the inversion effect of biomechanically possible hand posture identification appeared earlier than that of biomechanically impossible hand posture identification in terms of RT results, demonstrating that the lifetime experience (of biologically possible postures) affected the object discrimination inversion effect.

## Experiment 2

The hand posture inversion effect was not shown in earlier blocks, but it occurred in blocks 4 (only for biomechanically possible hand postures) and 5 (for both biomechanically possible and impossible hand postures) in Experiment 1. In other words, there was little initial superiority for any specific orientation in hand posture recognition, but after repeated exposure, it is likely that the canonical representation of upright orientations became accessible and consequently, the inversion effect became evident. Note that in Experiment 1, both upright and inverted hands were exposed equally, yet the inversion effect was shown most likely due to greater lifetime exposure to upright hands. However, Experiment 1 could not answer whether the naturally formed hand posture inversion effect was robust and stable, or just happened by chance. Hence, we designed an experiment to further explore this question. Specifically, the design of Experiment 2 was identical to that of Experiment 1, except that each participant repeated the tests in Experiment 1 for three consecutive days. In this way, we could analyze the data from day 1 to day 3 to observe whether the hand posture inversion effect was significant and stable. Thus, we hypothesized that since the hand posture inversion effect was formed on day 1, it would remain stable for the following two days.

### Method

*Participants.* Thirty-one undergraduate students of Chaohu University, China (16 men and 15 women, average age of 19.87 years) took part in Experiment 2; they had no such prior experience, and each received a gift for their participation. All of them were healthy, right-handed, and had normal or corrected-to-normal vision. The study was reviewed and approved by the Institutional Review Board of the Human Research Ethics Committee of the University.

*Stimuli.* The stimuli and apparatus were identical to that in Experiment 1.

*Experiment design*. The design of the experiment was a 3 (day: 1, 2, 3) × 5 (block: 1, 2, 3, 4, and 5) × 2 (match: matched pair and mismatched pair) × 2 (types of hand postures: biomechanically possible and biomechanically impossible) × 2 (orientation: upright and inverted hand postures) factorial design.

### Procedure

The procedure was the same as that in Experiment 1, except that the experiment was repeated and lasted for three days. All participants tested together in a computer room and were required to complete the five blocks identical to Experiment 1 each day. The start time of the experiment was 8 p.m. every day. Both RTs and error rates were recorded.

### Data Analysis

Only the data for identical pairs (matched pairs) were analyzed, and the data for different pairs (mismatched pairs) were discarded. RTs beyond three standard deviations from the mean were removed (0.21% of trials were discarded). Error rates and RTs were analyzed. A 5 (block: 1, 2, 3, 4, 5) × 2 (types of hand postures: biomechanically possible hand postures and biomechanically impossible hand postures) × 2 (orientation: upright hand postures and inverted hand postures) three-way repeated-measures ANOVA was carried out for data in each of the three days.

We then compared the magnitude of the inversion effect (the error rate of the inverted hand posture minus that of the upright hand posture and the RT of the inverted hand posture minus that of the upright hand posture) over three days and conducted a 3 (day: 1, 2, 3) × 2 (types of hand postures: biomechanically possible hand postures and biomechanically impossible hand postures) repeated-measures ANOVA.

### Results

*Error rate.* For the error rates on the first day, a three-way repeated-measures ANOVA was performed. The results showed that there was no main effect of orientation [*F*(1, 30) = 0.163, *P* = .689] and type of hand posture [*F*(1, 30) = 0.074, *P* = .787], although the general average trend of error rates demonstrated a lower error rate for upright hand postures (6.0%) compared with inverted hand postures (6.3%). The main effect of the block was significant [*F*(4, 120) = 4.564, *P* = .002]. The interaction effect between the block and orientation was significant [*F*(4, 120) = 4.602, *P* = .002]; the other two-way interaction effects and three-way interaction effects were not significant.

Further analysis revealed that, for block 2, the error rate of upright hand postures (8.1%) was significantly larger than that of inverted hand postures (4.8%) [*F*(1, 30) = 4.59, *P* = .040]. For block 3, the error rate of upright hand postures (3.2%) was significantly smaller than that of inverted hand postures (7.2%) [*F*(1, 30) = 12.29, *P* = .001]. While for blocks 1, 4, and 5, there were no significant difference between upright hand postures (8.7%, 5.3%, and 4.8%) and inverted hand postures (10.1%, 4.4%, and 5.0%) [*F*(1, 30) = 0.96, *P* = .335; *F*(1, 30) = 0.58, *P* = .453; *F*(1, 30) = 0.02, *P* = .890, respectively].

For the error rates on the second day, there was a significant main effect of orientation [*F*(1, 30) = 8.09, *P* = .008], showing a lower error rate for upright hand postures (2.5%) compared with inverted hand postures (4.3%). There was no main effect of the type of hand posture [*F*(1, 30) = 1.363, *P* = .252] and block [*F*(4, 120) = 1.496, *P* = .208]. There were no significant two-way or three-way interaction effects.

For the error rates on the third day, there was a significant main effect of orientation [*F*(1, 30) = 5.642, *P* = .024], which showed a lower error rate for upright hand postures (1.5%) compared with inverted hand postures (2.8%). There was no main effect of the type of hand posture [*F*(1, 30) = 1.013, *P* = .322] and block [*F*(4, 120) = 1.065, *P* = .377]. The interaction effect between the type of hand posture and orientation was significant [*F*(1, 30) = 6.784, *P* = .014]. The other two-way and three-way interaction effects were not significant.

**Figure 5.**
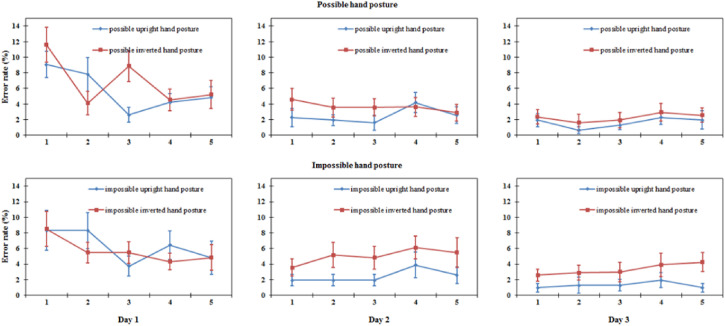
Error rates of the upright and inverted condition in each block over three days for the biomechanically possible hand postures and biomechanically impossible hand postures in the three-day same/different task in Experiment 2. Error bars represent the standard errors of the means for each condition.

Further analysis revealed that, for biomechanically possible hand postures, the error rate for upright hand postures (1.6%) was slightly lower than that for inverted hand postures (2.3%), although the difference between them was not significant [*F*(1, 30) = 1.42, *P* = .243]. For biomechanically impossible hand postures, the error rate for upright hand postures (1.3%) was also lower than that for inverted hand postures (3.3%), and the difference between the two postures was significant [*F*(1, 30) = 8.81, *P* = .006] (see Figure 5).

A 3 (day: 1, 2, 3) × 2 (types of hand postures: biomechanically possible and biomechanically impossible) two-way repeated-measures ANOVA, with the difference between the error rate of inverted orientation and that of upright orientation as a dependent variable, was carried out. The results indicated that the main effect of the day did not reach significance [*F*(2, 60) = 3.017, *P* = .056], but showed that the inversion effect was the lowest on day 1 (0.3%), whereas the inversion effect on day 2 was close to that on day 3 (1.8% and 1.3%, respectively). Neither the main effect of the type of hand posture nor the interaction effect between the two was significant.

*Reaction time.* A three-way repeated-measures ANOVA was carried out for the RTs on the first day. The main effect of orientation was marginally significant [*F*(1, 30) = 3.95, *P* = .056], showing faster RTs for upright hand postures (635 ms) compared with inverted hand postures (645 ms). There was a significant main effect of block [*F*(4, 120) = 12.889, *P* < .001] and type of hand posture [*F*(1, 30) = 6.027, *P* = .020]. There were no significant two-way or three-way interaction effects.

On the second day, the main effect of orientation was significant [*F*(1, 30) = 23.801, *P* < .001], showing faster RTs for upright hand postures (543 ms) compared with inverted hand postures (562 ms). There was also a significant main effect of block [*F*(4, 120) = 6.649, *P* < .001] and type of hand posture [*F*(1, 30) = 15.795, *P* < .001]. The interaction effect between block and orientation was significant [*F*(4, 120) = 2.938, *P* < .05]. The other two-way or three-way interaction effects were not significant.

Further analysis revealed that, for blocks 1 and 2, RTs for upright hand postures (568 and 555 ms) were slightly faster than those for inverted hand postures (576 and 567 ms), whereas the differences between them were not significant [*F*(1, 30) = 0.92, *P* = .346; *F*(1, 30) = 2.76, *P* = .107]. In contrast, for blocks 3, 4, and 5, RTs for upright hand postures (549, 530, and 512 ms) were significantly faster than those for inverted hand postures (566, 551, and 549 ms) [*F*(1, 30) = 5.87, *P* = .022; *F*(1, 30) = 12.14, *P* = .002; *F*(1, 30) = 28.46, *P* < .001, respectively].

**Figure 6.**
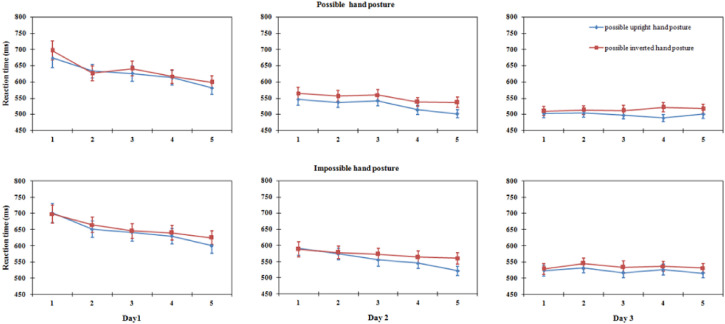
Reaction time in the upright and inverted orientation in five blocks over three days for the biomechanically possible and biomechanically impossible hand postures in the three-day same/different task in Experiment 2. Error bars represent the standard errors of the means for each condition.

On the third day, the main effect of orientation was significant [*F*(1, 30) = 11.945, *P* = .002], showing faster RT for upright hand postures (511 ms) compared with inverted hand postures (525 ms). The main effect of type of hand posture was significant [*F*(1, 30) = 16.404, *P* < .001], but the main effect of the block was not significant [*F*(4, 120) = 0.347, *P* = .845]. No two-way or three-way interaction effects were significant (see Figure 6).

A 3 (day: days 1, 2, 3) × 2 (types of hand postures: biomechanically possible and biomechanically impossible) two-way repeated-measures ANOVA, with the difference between RT of inverted orientation and that of upright orientation as a dependent variable, was carried out. The results showed that the main effect of a particular day was not significant [*F*(2, 60) = 1.545, *P* = .222]. Although it demonstrated that the inversion effect was the lowest on day 1 (11 ms), the inversion effect on day 2 was close to that on day 3 (19 and 14 ms, respectively). Neither the main effect of the type of hand posture nor the interaction effect between the two was significant.

### Discussion

Experiment 2 further examined whether the hand posture identification inversion effect was naturally yielded after repeated trials and remained stable. As the RT results show, throughout day 2 the inversion effects were stable. These results suggest that the hand posture inversion effect did not yield randomly and the processing mode of participants was changed from initially depending on feature detection to configural processing. In other words, these results suggest experience would lead to a transformation process of object recognition processing mode, from feature detection to a stable configural processing, further supporting the configural processing continuum hypothesis. In addition, during the three-day discrimination task, it is possible that once the participants selected the processing mode, they would not choose another processing mode in the same paradigm for the sake of convenience.

In Experiment 2, the inversion effect was initially elicited on the second day. That is, compared with Experiment 1, the inversion effect yielded slightly later in Experiment 2, most likely reflecting individual differences. However, the general trend of late-onset of the inversion effect was consistent across the two experiments.

## Experiment 3

Previous studies showed that the body inversion effect was robust (Reed et al., 2003, 2006; Tao & Tao, 2018). In Experiment 3, we aimed to explore whether the hand postures would elicit an inversion effect consistently across different tasks, such as an identification task (Husk et al., 2007), and yield an earlier and larger effect in contrast to the same/different paradigm in Experiments 1 and 2.

### Method

*Participants*. Twenty-six undergraduate students of Chaohu University, China (16 men and 10 women, average age, 20.7 years) participated in the experiment; none of them had participated in similar experiments before and each received a gift after participation. All were healthy, right-handed, and had normal or corrected-to-normal vision. The study was reviewed and approved by the Institutional Review Board of the Human Research Ethics Committee of the University.

**Figure 7.**
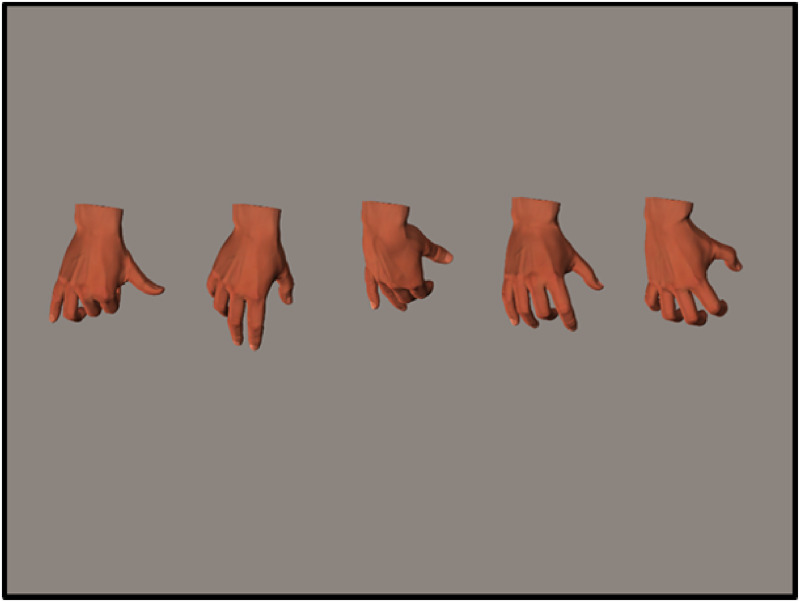
Examples of hand posture stimuli in the identification task in Experiment 3.

*Stimuli*. The stimuli in Experiment 3 were identical to those in Experiment 1. Nothing was changed in the hand postures; only the stimuli were resized to 160 × 213 pixels. In the presentation of the second stimuli in each trial, instead of one hand stimulus as in previous experiments, five biomechanically possible hand postures or five biomechanically impossible hand postures were presented simultaneously; these were fixed in the same order and position in each trial. The visual angle of each hand posture was <3° (see Figure 7).

### Experiment Design

The design of the experiment was a 5 (block: 1, 2, 3, 4, and 5) × 2 (types of hand postures: biomechanically possible and biomechanically impossible) × 2 (orientation: upright and inverted hand postures) factorial design. The experiment consisted of five blocks, with 400 trials in total. The order of the stimulus presentation, types of hand postures, and orientations were completely randomized. Notably, the first and second pictures presented were consistent in hand posture type and orientation.

**Figure 8.**
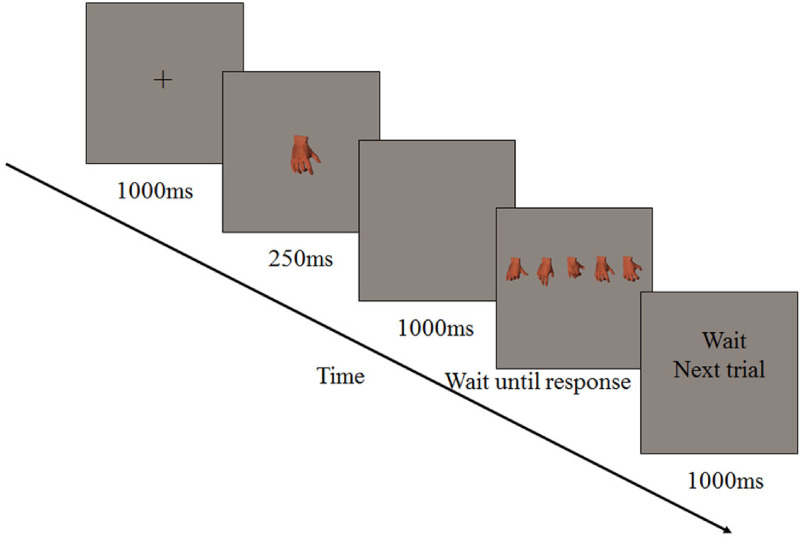
The sequence of events during a trial in Experiment 3.

*Procedure*. The formal experimental procedure was compiled by E-Prime 1.1. The monitor was 17″ with a refresh rate of 75 Hz and a 1,024 × 768-pixel resolution. The participants were tested together in a computer room. In each trial, a fixation was first presented for 1,000 ms, followed by a stimulus lasting 250 ms. A blank screen was then presented for 1,000 ms. Next, five pictures were presented and they remained on the screen until the participant responded. When the second stimulus was presented, the participants were asked to select from the five pictures, a picture of the hand posture identical to the first picture presented just before, move the mouse over the hand posture, and select to confirm (see Figure 8).

At the beginning of each trial, the software automatically moved the mouse to the center of the screen. Each participant was required to move the mouse from the center to respond as per the instructions and respond as quickly as possible without compromising accuracy. Both error rates and RTs were recorded. Eight practice trials were conducted prior to the formal experiment. Each participant spent about 40 min completing the experiment.

### Data Analysis

The error rates and RTs were analyzed, but RTs beyond three standard deviations from the mean were removed (2.7%, 284 trials were discarded). A 5 (block: 1, 2, 3, 4, and 5) × 2 (types of hand postures: biomechanically possible and biomechanically impossible) × 2 (orientation: upright and inverted hand postures) three-way repeated-measures ANOVA was carried out.

### Results

**Figure 9.**
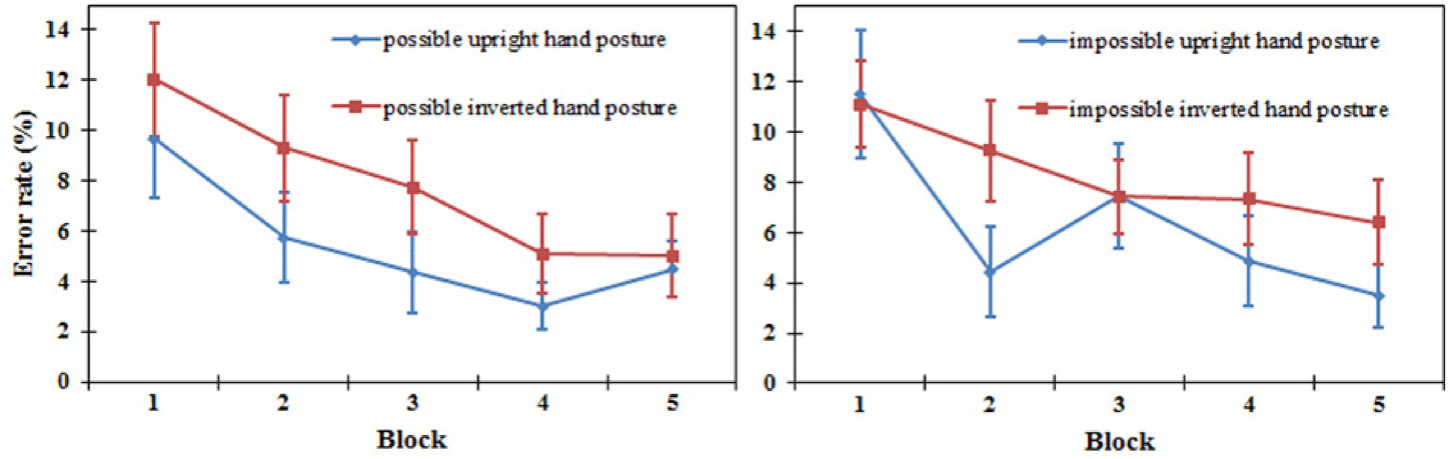
Error rates of the upright and inverted conditions in each block for the biomechanically possible hand postures (left) and biomechanically impossible hand postures (right) in the identification task in Experiment 3. Error bars represent the standard errors of the means for each condition.

*Error rate.* For the error rates, a three-way repeated-measures ANOVA was performed. The results showed a significant main effect of orientation [*F*(1, 25) = 13.771, *P* = .001], and the general trend revealed a lower error rate for upright hand postures (5.9%) compared with inverted hand postures (8.1%). The main effect of type of hand posture was not significant [*F*(1, 25) = 1.115, *P* = .301]. The main effect of block was significant [*F*(4, 100) = 11.761, *P* < .001]. There were no significant two-way or three-way interaction effects (see Figure 9).

*Reaction time.* For the RTs, a three-way repeated-measures ANOVA was performed. The results showed that there was a significant main effect of orientation [*F*(1, 25) = 8.632, *P* < .01], whose general trend revealed a quicker RT for upright hand postures (1,152 ms) compared with inverted hand postures (1,188 ms). The main effect of the type of hand posture was not significant [*F*(1, 25) = 1.989, *P* = .171]. The main effect of the block was significant [*F*(4, 100) = 62.203, *P* < .001]. The interaction effect between the type of hand posture and orientation was significant [*F*(1, 25) = 17.290, *P* < .001]. Other two-way or three-way interaction effects were not significant.

**Figure 10.**
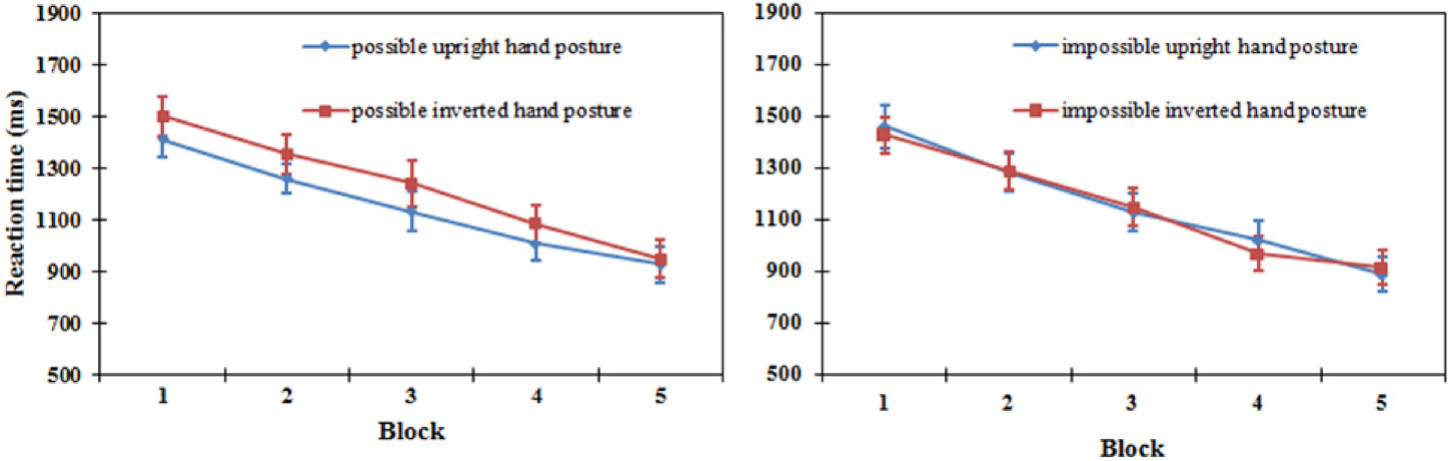
Reaction time in the upright and inverted direction in each block for the biomechanically possible hand postures (left) and biomechanically impossible hand postures (right) in the identification task in Experiment 3. Error bars represent the standard errors of the means for each condition.

Further analysis showed that, for biomechanically possible hand postures, RTs for upright hand postures (1,148 ms) were faster than those for inverted hand postures (1,227 ms), and the difference between them was significant [*F*(1, 25) = 18.98, *P* < .001]. For biomechanically impossible hand postures, there was no significant difference between upright hand postures (1,156 ms) and inverted hand postures (1,150 ms) [*F*(1, 25) = 0.26, *P* = .613] (see Figure 10).

### Discussion

Using the identification task developed by Husk et al. (2007), Experiment 3 examined whether the hand posture inversion effect was consistent across different tasks. For biomechanically possible hand postures, both RTs and error rates showed a significant inversion effect, revealing that upright hand postures were distinguished faster and more accurately than inverted ones. The results suggested that the hand posture inversion effect had cross-task consistency. Moreover, for biomechanically possible hand postures (but not for biomechanically impossible hand postures), a robust inversion effect was found. In addition, it appears that, compared to Experiments 1 and 2, the onset of the inversion effect produced in the identification task in Experiment 3 was much earlier. A number of reasons could be responsible for the earlier onset. Compared to the same/different discrimination task in Experiment 1, the identification task in Experiment 3 involved increased exposure to stimuli (in the second presentation in each trial), improving participants’ experience with the hand postures. Moreover, comparisons between multiple stimuli in the second presentation might be easier for the strategy of configural processing.

In addition, in this experiment, the pattern of results for biomechanically possible hand postures appeared to be much more robust than that of the results for biomechanically impossible hand postures. This further supports the view that the effect of prior lifetime exposure might interact with the effect of short-term exposure during the experiment that triggers the inversion effect.

## Experiment 4

In Experiments 1, 2, and 3, with the increased exposure to hand postures (even with upright and inverted orientations presented equally throughout the experiments), the inversion effect formed naturally. However, compared to the body inversion effect (Tao & Sun, 2013), the hand posture inversion effect was much smaller likely because the difference in exposure between two orientations was smaller than that of body posture. After all, humans often see their own or others' hands in daily life, which can be represented in a variety of gestures and orientations; thus, they are somewhat familiar with both upright and inverted hand postures. Hence, here we used a training procedure (similar to the task in Experiment 3) to explore whether hand posture recognition would yield an inversion effect based on the training orientation and to examine the flexibility of the hand posture processing mode. We hypothesized that in the posttest, the participants in the upright hand posture training group could recognize the upright hand posture faster than in the inverted hand posture. Likewise, participants in the inverted hand posture training group could recognize the inverted hand posture faster than the upright hand posture.

### Method

*Participants*. Twenty undergraduate students of Chaohu University, China (10 men and 10 women, average age, 21.3 years) participated in the experiment; each received RMB 5 Yuan as a reward after the experiment. None of them had participated in similar experiments. All participants were healthy, right-handed, and had normal or corrected-to-normal vision. The study was reviewed and approved by the Institutional Review Board of the Human Research Ethics Committee of the University.

*Stimuli*. The stimuli were identical to those in Experiment 1. However, only biomechanically possible hand postures were selected for use as experimental materials to reduce the workload of the participants.

*Experimental design.* The experiment was divided into two stages. The first part was the training stage when 20 participants were randomly divided into two groups of 10 participants (5 men, 5 women), who underwent the training for upright hand postures and inverted hand postures, respectively. The two groups subsequently underwent a posttest following training.

The factors in the training task included a 5 (block: 1, 2, 3, 4, and 5) × 2 (training group: upright orientation and inverted orientation) design, and the training group is the between-participant factor. Each block of the experiment consisted of 80 trials, including 10 hand postures (5 left-hand postures and five right-hand postures).

The posttest adopted a 2 (trained group: upright orientation and inverted orientation trained groups) × 2 (stimulus orientation: upright and inverted) factorial design. The experiment had two blocks. Each block consisted of 80 trials, including 40 upright trials and 40 inverted orientation trials.

*Procedure.* The formal experimental procedure was compiled by E-Prime 1.1. The monitor was 17″ with a refresh rate of 75 Hz and a 1,024 × 768-pixel resolution. The participants were tested together in the computer room, seated at 70 cm from the monitor. The center of the computer screen was placed at eye level by adjusting the height of the chair.

In the training stage, each trial began with a fixation for 1,000 ms, followed by a stimulus lasting 250 ms. A blank screen was then presented for 1,000 ms. Next, the second stimuli were presented, which remained on the screen until the participant responded. Then software automatically gave feedback and displayed that for 1,000 ms after the response of the participants, indicating whether the response was correct or incorrect. Then, the reminder of the subsequent stimulus presentation was displayed for 1,000 ms.

At the beginning of each trial, the software automatically moved the mouse to the center of the screen. Each participant was asked to move the mouse from the center and respond per the instructions as quickly as possible without compromising accuracy. The training procedure was similar to the identification task in Experiment 3; as in Experiment 3, participants were instructed to choose a picture identical to the first picture presented just before from the five picture array, then move the mouse over the chosen picture and select to confirm. The procedure of the posttest stage was identical to that of the training stage, except that the feedback was removed.

### Data Analysis

For the training data, the error rates and RTs were analyzed. RTs beyond three standard deviations from the mean were discarded (1.21% of trials were discarded). A 5 (block: 1, 2, 3, 4, and 5) × 2 (trained group: upright orientation and inverted orientation) mixed ANOVA was carried out.

For the posttest, error rates and RTs were analyzed. RTs beyond three standard deviations from the mean were discarded (0.71% of trials were discarded). A 2 (trained group: upright orientation and inverted orientation trained groups) × 2 (stimulus orientation: upright and inverted) mixed ANOVA was carried out.

### Results

**Figure 11.**
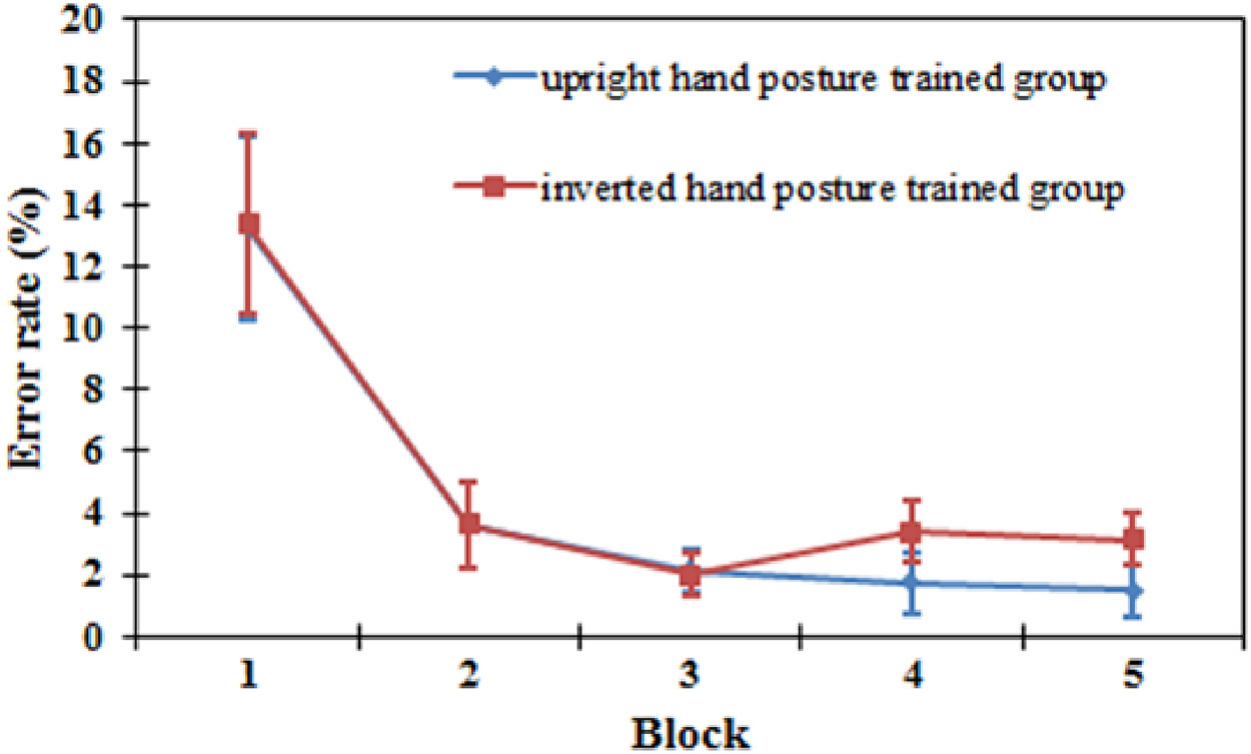
Error rates in each block in the training task for the upright hand posture training group and the inverted hand posture training group. Error bars represent the standard errors of the means for each condition.

*Error rate.* For the training task, the error rates were analyzed in a 5 (block: 1, 2, 3, 4, and 5) × 2 (training group: upright orientation and inverted orientation) mixed ANOVA. The results showed that there was a significant main effect of block (*F* (4, 72) = 23.809, *P* < .001), indicating that with the increase in blocks, the error rates decreased across blocks (block 1: 13.3%; block 2: 3.6%; block 3: 2.1%; block 4: 2.6%; block 5: 2.3%; see Figure 11). The main effect of the training group was not significant [*F*(1, 18) = 0.210, *P* = .652]. The interaction effect of block and orientation was not significant [*F*(4, 72) = 0.206, *P* = .934].

**Figure 12.**
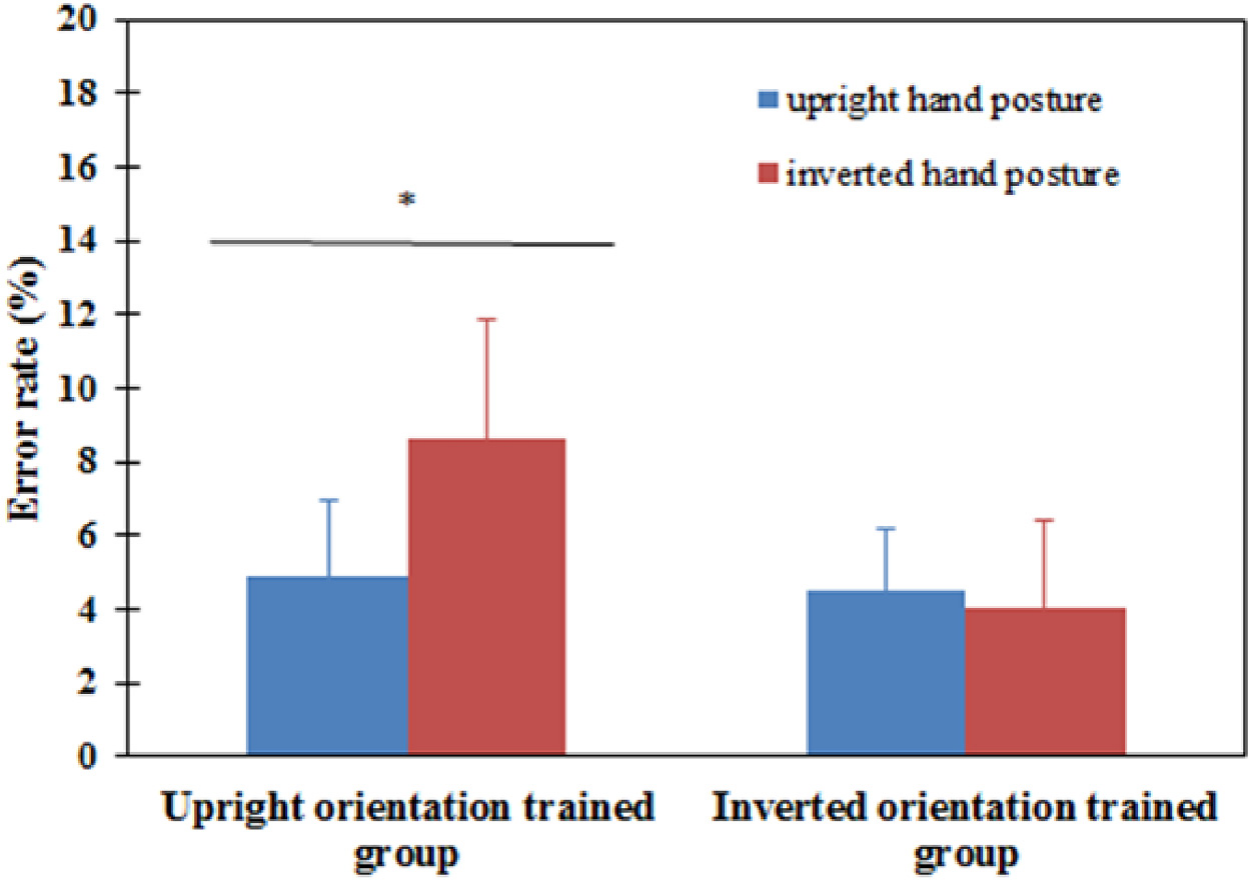
Error rates of upright and inverted conditions for each block in the posttest for the upright orientation trained group and the inverted orientation trained group in Experiment 4. Error bars represent the standard errors of the means for each condition. *P < .05, statistically significant.

For posttest, a mixed ANOVA was carried out to analyze error rates. The results showed that there was no significant main effect on the trained group [*F*(1, 18) = 0.591, *P* = .452] and stimulus orientation [*F*(1, 18) = 2.233, *P* = .152]. However, the interaction between the trained group and stimulus orientation was marginally significant [*F*(1, 18) = 3.819, *P* = .066]. Simple effect analysis showed that in the upright orientation trained group, the upright hand posture performance was significantly more accurate than that of the inverted hand posture [*F*(1, 18) = 5.59, *P* = .025]. However, in the inverted orientation trained group, the inverted hand posture performance was not significantly more accurate than that of the upright hand posture [*F*(1, 18) = 0.11, *P* = .749] (see Figure 12).

**Figure 13.**
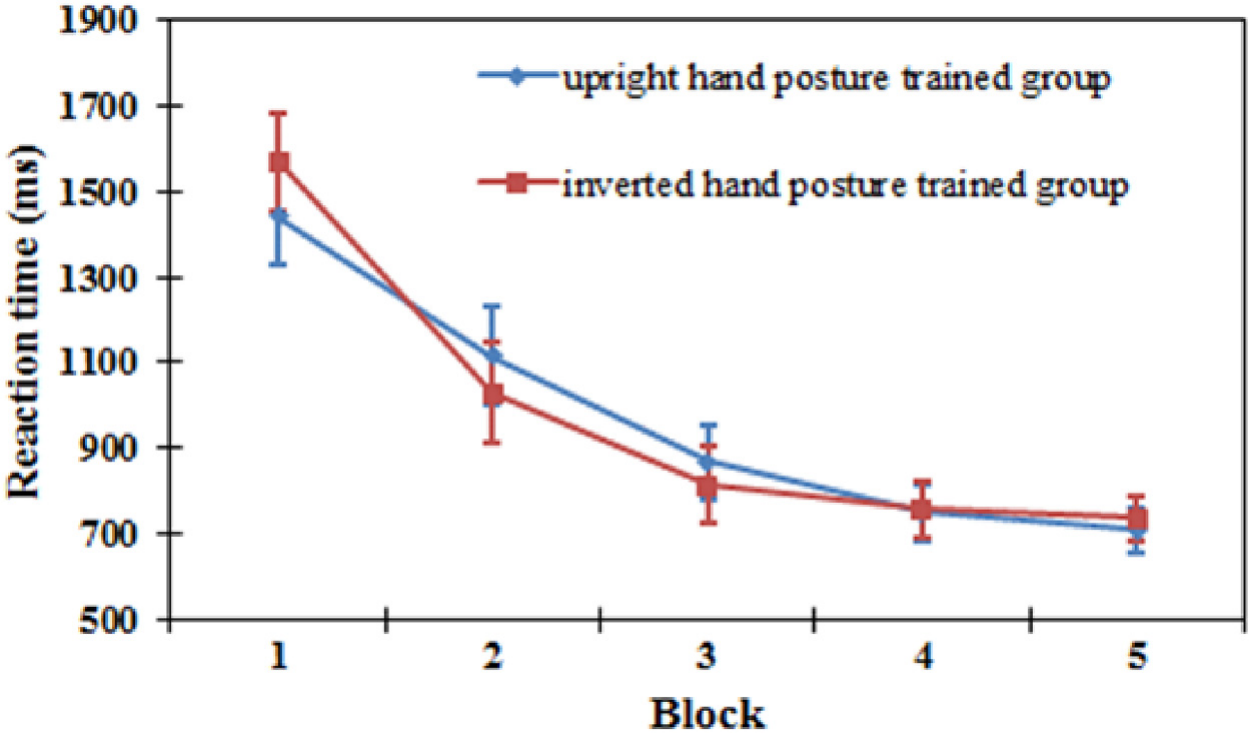
Reaction time in each block in the training task for the upright hand posture training group and the inverted hand posture training group in Experiment 4. Error bars represent the standard errors of the means for each condition.

*Reaction time.* The results showed that there was a significant main effect of the block [*F*(4, 72) = 77.409, *P* < .001], indicating that with increasing blocks, the RTs became faster (block 1: 1,508 ms; block 2: 1,073 ms; block 3: 841 ms; block 4: 753 ms; block 5: 724 ms; see Figure 13). The main effect of orientation was not significant [*F*(1, 18) = 0.001, *P* = .971]. The interaction effect between the two was not significant [*F*(4, 72) = 1.238, *P* = .302] (see Figure 13).

**Figure 14.**
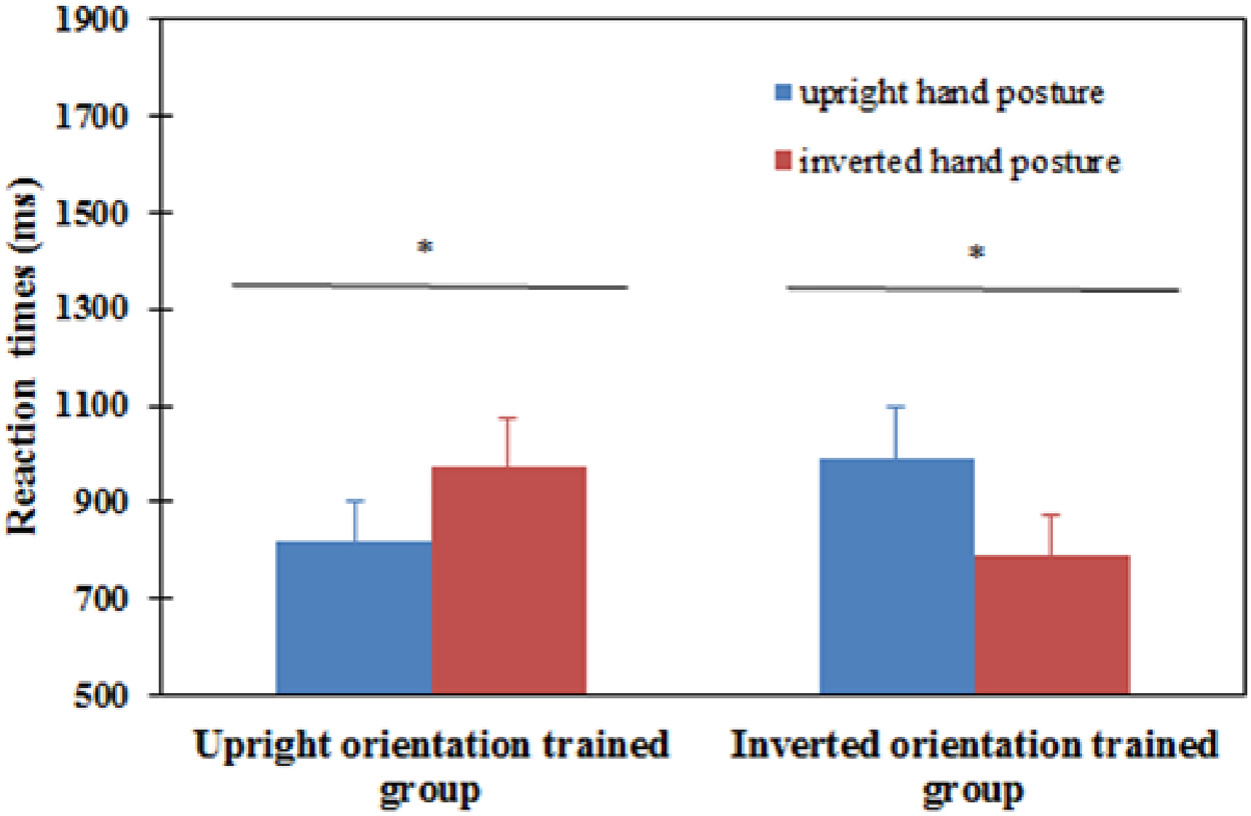
Reaction time of upright and inverted conditions for each block in the posttest for the upright orientation trained group and the inverted orientation trained group in Experiment 4. Error bars represent the standard errors of the means for each condition. *P < .05, statistically significant.

For posttest, a mixed ANOVA was carried out to analyze RTs. The results showed that there was no significant main effect on the trained group [*F*(1, 18) = 0.001, *P* = .971] and stimulus orientation [*F*(1, 18) = 0.256, *P* = .619]. However, the interaction between the trained group and stimulus orientation was significant [*F*(1, 18) = 13.644, *P* < .01]. Simple effect analysis showed that in the upright orientation trained group, the upright hand posture was recognized significantly faster than the inverted hand posture [*F*(1, 18) = 5.08, *P* = .037]. In the inverted orientation trained group, the inverted hand posture was recognized significantly faster than the upright hand posture [*F*(1, 18) = 8.82, *P* = .008] (see Figure 14).

### Discussion

The results of the hand posture discrimination from Experiments 1 and 2 demonstrated a shift in processing mode from an initially absent inversion effect to a later robust effect. However, participants received comparable exposure to upright and inverted hand postures during the experiments. Therefore, the inversion effect should not appear because they have developed a greater canonical representation of upright hand postures simply from experience gained during the experiment. Rather slightly greater lifetime exposure to upright hand postures might contribute to the inversion effect.

As an alternative, artificial training could be another way to influence the inversion effect. In Experiment 4, we utilized a training task to expose participants to upright or inverted hand posture orientations. With the increase in the participants’ experience of upright or inverted hand postures, they would display the hand inversion effect in the posttest based on their specific training orientation. Indeed, Experiment 4’s posttest results support our hypothesis, revealing that both training groups developed a hand posture inversion effect based on their specific training orientation, and provided direct evidence for the perspective that different levels of exposure in a particular orientation affect the specific object recognition inversion effect.

## General Discussion

Four experiments investigated the hand posture inversion effect by using the inverted paradigm with discrimination and identification tasks. The findings show the hand posture inversion effect was revealed only in the later test blocks, suggesting a change in the upright hand posture processing mode occurred as participants acquired task experience. It was also found that the hand posture inversion effect was stable after it was established. This effect remained consistent across different tasks, although the exact onset time and magnitude was variable. Furthermore, following differential exposure of upright or inverted hand postures administered via artificial training, the hand posture inversion effect was developed corresponding to the specific training orientation.

The present study demonstrated the unique late emergence of the hand posture inversion effect. Specifically, the RT results of Experiments 1 and 2 revealed the hand posture inversion effect developed from an absence of the effect early on to a significant robust effect later in the experiment. This demonstrates the flexibility of hand recognition processing. Participants might have mainly relied on feature detection processing in earlier blocks and configural processing in later blocks for upright hand postures, which is consistent with the configural processing continuum hypothesis (Reed et al., 2006; Tao & Tao, 2018). In other words, with the increased exposure, the participants automatically selected a more efficient processing method to distinguish the hand postures in a specific direction. This further supports the idea that feature detection and configural processing are not different in nature but are rather different in quantity. If feature detection and configural processing have substantial differences in quality, the participants will not be able to change their processing modes quickly and naturally, especially not within five experimental blocks.

The results of Experiments 1 and 2 on hand posture processing were inconsistent with previous research by Tao and Sun (2013) involving body posture stimuli. The most likely reason for this distinction is the difference in daily exposure between object orientations. In other words, the participants had only slightly more experience with upright hand postures than that with inverted ones, while the difference between upright body postures and inverted body postures was considerable, namely, the exposure to body postures for the participants was almost always upright. Thus, unlike body postures, the participants did not form a canonical orientation memory of upright hand postures, which, at first, did not significantly affect the standard viewpoint of hand posture.

The findings also show that the hand inversion effect was stable and consistent across tasks. Specifically, we found that when the procedure in Experiment 1 was repeated throughout three days in Experiment 2, the hand posture inversion effect, formed on day 1, remained constant on days 2 and 3, replicating the findings of Experiment 1. In the identification paradigm in Experiment 3, a hand posture inversion effect was also generated, indicating that the hand posture inversion effect can be revealed across different paradigms. Similar to previous studies of the body inversion effect (Reed et al., 2003; Tao & Sun, 2013), although the hand posture inversion effect tended to be evident only in later blocks, it was stable and consistent across different tasks.

Our results also support the notion that the inversion effect is not limited to face-like or body-like objects, but hand posture recognition also shows an inversion effect. Such hand posture inversion effects can also be explained by the expertise hypothesis (Diamond & Carey, 1986; Gauthier & Tarr, 1997; Gauthier et al., 1999, 2000). As stated in the introduction, the expertise hypothesis suggests that face object recognition processing could be akin to that of nonface objects. This would indicate that face recognition is not a special processing, but a form-general mechanism, which could also be engaged by hand posture discrimination and hand posture identification.

Unlike faces and body postures where people have considerably more experience with upright orientations compared to inverted orientations, exposure to inverted hands is more comparable to upright hand postures in daily life. Thus, participants may not have developed a robust canonical orientation memory of upright hand postures. In Experiment 4, using the methods in the research by Gauthier and Tarr (1997) and Husk et al. (2007), the differences in experience contributed to different processing styles for upright versus inverted orientation were manipulated by training. In other words, as the amount of exposure to the upright or inverted direction increased, the participants gradually produced the hand posture inversion effect based on their specific training orientation. The most probable reason for this is the difference in recent exposure in object orientations became the decisive factor for the occurrence of the inversion effect in object recognition. For error rate and response time, the pattern of results of the upright and the inverted hand posture training groups was almost comparable in overall performance, yet formed symmetrical orientation selectivity. Participants might have regarded the training orientation as the “upright” orientation, while the nontraining orientation as the “inverted.” Hence, the participants empirically considered the training orientation as their canonical orientation and adopted configural processing to complete the tasks.

The contention between the face processing-specificity hypothesis and the expertise hypothesis is whether the brain mechanism of object recognition processing is single-module or multiple-module. However, there is substantial research providing behavioral and electrophysiological evidence that the brain mechanisms of object recognition processing are a form-general mechanism, demonstrated in the activation of N170 (Stekelenburg & de Gelder, 2004) and fusiform face area (Gauthier et al., 1999) in the recognition task by domain experts. The current study shows a rapid transition from feature detection processing to holistic processing, supporting brain plasticity and cognitive flexibility. Specifically, although we did not have electrophysiological data, the behavioral results are consistent with the notion that the hand posture inversion effect is easy to show (even in the reversal of orientations), which suggested that both holistic processing and feature detection processing can be used in hand posture recognition and the hand posture identification paradigm.

In this study, the causes of the hand posture inversion effect and processing modes in hand posture recognition were explored from the perspective of exposure differences among hand orientations. According to these results, three conclusions were drawn: (1) the difference in long-term exposure to specific object orientations is the crucial factor for the appearance of the hand posture inversion effect; (2) the difference in recent exposure in object orientations can also determine the type of processing mode; and (3) the processing mode for upright hand postures and that for inverted hand postures are not qualitatively different.

Nevertheless, some limitations in this study exist. First, although our results suggest that differences in long-term exposure in specific object orientations might be attributable to the inversion effect, further studies need to be performed to provide direct evidence for this explanation. Second, our explanations for the results were drawn based on behavioral results. Explorations using ERP, functional magnetic resonance imaging, and other brain imaging methodologies could be implemented to provide complimentary information revealing the neurological basis of the hand inversion effect. Additionally, the paradigm used in the current study would be ideal in studies of brain mechanisms where the visual stimulus is identical, but the change in processing mode is evident over a shorter period of time.
